# Are Honey Bees at Risk from Microplastics?

**DOI:** 10.3390/toxics9050109

**Published:** 2021-05-15

**Authors:** Yahya Al Naggar, Markus Brinkmann, Christie M. Sayes, Saad N. AL-Kahtani, Showket A. Dar, Hesham R. El-Seedi, Bernd Grünewald, John P. Giesy

**Affiliations:** 1 Zoology Department, Faculty of Science, Tanta University, Tanta 31527, Egypt; 2 General Zoology, Institute for Biology, Martin Luther University Halle-Wittenberg, 06120 Halle, Germany; 3 School of Environment and Sustainability, University of Saskatchewan, Saskatoon, SK S7N 5C8, Canada; markus.brinkmann@usask.ca; 4 Global Institute for Water Security, University of Saskatchewan, Saskatoon, SK S7N 3H5, Canada; 5 Toxicology Centre, University of Saskatchewan, Saskatoon, SK S7N 5B3, Canada; jgiesy@aol.com; 6 Department of Environmental Sciences, Baylor University, Waco, TX 76798-7266, USA; christie_sayes@baylor.edu; 7 Laboratory of Bio-Control and Molecular Biology, Department of Arid Land Agriculture, College of Agricultural and Food Sciences, King Faisal University, Hofuf 31982, Saudi Arabia; salkahtani@kfu.edu.sa; 8 Division of Agricultural Entomology, KVK-Kargil II, Sher-e-Kashmir University of Agricultural Sciences and Technology of Kashmir, Srinagar 191111, India; showketdar43@gmail.com; 9 International Research Center for Food Nutrition and Safety, Jiangsu University, Zhenjiang 212013, China; hesham.el-seedi@fkog.uu.se; 10 Pharmacognosy Group, Department of Pharmaceutical Biosciences, Biomedical Centre, Uppsala University, 751 23 Uppsala, Sweden; 11 Department of Chemistry, Faculty of Science, Menoufia University, Shebin El-Kom 32512, Egypt; 12 Institut für Bienenkunde, Polytechnische Gesellschaft Frankfurt am Main, Goethe-Universität, 61440 Oberursel, Germany; b.gruenewald@bio.uni-frankfurt.de; 13 Center for Integrative Toxicology, Department of Zoology, Michigan State University, East Lansing, MI 48824, USA

**Keywords:** microplastics, honey bees, ecological risk assessment, environmental pollution, pollinator crisis

## Abstract

Microplastics (MPs) are ubiquitous and persistent pollutants, and have been detected in a wide variety of media, from soils to aquatic systems. MPs, consisting primarily of polyethylene, polypropylene, and polyacrylamide polymers, have recently been found in 12% of samples of honey collected in Ecuador. Recently, MPs have also been identified in honey bees collected from apiaries in Copenhagen, Denmark, as well as nearby semiurban and rural areas. Given these documented exposures, assessment of their effects is critical for understanding the risks of MP exposure to honey bees. Exposure to polystyrene (PS)-MPs decreased diversity of the honey bee gut microbiota, followed by changes in gene expression related to oxidative damage, detoxification, and immunity. As a result, the aim of this perspective was to investigate whether wide-spread prevalence of MPs might have unintended negative effects on health and fitness of honey bees, as well as to draw the scientific community’s attention to the possible risks of MPs to the fitness of honey bees. Several research questions must be answered before MPs can be considered a potential threat to bees.

## Background

Due to their potential for harming humans, animals, and the environment in general, microplastics (MPs) are emerging pollutants of concern that have received increasing attention in the last decade [1,2]. Results of recent studies have shown that MPs are ubiquitous in various environmental matrices, including air, soil, and water [3]. Some estimates are that humans consume as much as 52,000 particles per year; even more alarming are estimates of inhaled microplastics, which could be as much as 74,000 particles per year [4]. Microplastics are categorized as polymeric particles with a plasticizer component < 5 mm in diameter and are further classified into two subgroups, i.e., primary and secondary MPs. Those in the primary subgroup are produced directly as microscopic materials and are often intended to be used in consumer products, which include cosmetics, detergents, and cleaning products. Primary MPs are also commonly used as blast cleaning agents for dense materials, such as ship hulls in dockyards. Secondary MPs are produced when larger plastic materials degrade (break down) in the atmosphere or the aquatic environment due to natural weathering processes. For example, tire, road wear particles and municipal sewage sludge have been identified as potentially significant sources of microplastics in the environment [5,6]. Given the vast amount of MPs entering the environment, most MPs in the environment are thought to be secondary MPs [7,8].

Both primary and secondary MPs are ubiquitous and persistent pollutants, which have been detected in a wide variety of media, from soils to aquatic systems (e.g., wastewater treatment plant effluents, oceans, rivers, shorelines, and swamps) [9,10,11,12]. In addition, MPs have recently been found in urban, suburban, and even remote atmospheres far from their sources [13], which indicates possible long-distance atmospheric transport [14,15].

Recently, the wide-spread presence of MP contamination has drawn the attention of ecotoxicologists concerned about their potential toxicity [16]. Exposure of aquatic biota to MPs can induce toxicological effects, including lesser fitness, greater oxidative stress, immunological responses, and compromised intestinal function [17,18]. Additionally, an increasing body of evidence indicates that MPs interact with terrestrial organisms that mediate significant ecosystem services and functions, such as terrestrial fungi or several invertebrates e.g., pollinating insects [19]. Pollinators are inextricably linked to the natural environment and the production of foods; they maintain a genetically diverse angiosperm flora within most ecosystems and are, thus, essential for food crop pollination and human as well as livestock food security around the world.

European honey bees (*Apis mellifera*) play an important role in crop and wild plant pollination [20,21,22]. Considering the ecological and economic importance of honey bees, it is concerning that significant overwintering losses of honey bee colonies have been recorded in both Europe and the United States since 2006 [23,24]. Multiple biotic and abiotic factors, including parasites (ex. *Varroa destructor*), microbial infections, exposure to pesticides, loss of habitat and improper beekeeping practices, have been discussed as causes of these colony declines [25,26,27,28,29,30,31,32,33,34,35,36,37]. It has still not yet been studied, however, whether there is any potential link between exposure of honey bees to MPs and honey bee colony vitality. This assumption is not new; researchers have speculated that honey bee exposure to other emerging pollutants, such as environmental contaminants, metals and metal nanoparticles [38,39], and nanotechnology-based pesticides (NBPs) [40] could cause harm to the bees.

Tire wear and fragmented macroplastic that reaches the atmosphere by littering cause MP emissions in agricultural soils. Furthermore, farmers who use waste sludge and manure to fertilize their crops inadvertently add the microplastic particles contained in these biosolids to their crops [41,42]. In an analysis of macro- and microplastics in agricultural soil, researchers discovered up to 205 macroplastic parts per hectare (ha) and 0.34 to 0.36 microplastic particles per kilogram of soil (dry weight). Films and fragments made up the bulk of the plastic waste found, accounting for 91.36% of all macroplastic bits. Other shapes, such as rope, strapping tape, and textile wastes, were clustered together with an 8.64% contribution [43].

For decades, plastic particles were thought to be too large to move through the physical barriers of intact plant tissue. However, a recent study uncovered that microplastics, as well as their smaller counterparts termed “nanoplastics”, have potential to contaminate edible plants, including vegetables consumed by humans [44]. Those authors examined how crop plants (wheat (*Triticum aestivum*) and lettuce (*Lactuca sativa*)) absorb various microplastics from treated wastewater in hydroponic cultures, sand matrices, and sandy soil. The findings support the idea that submicrometre and micrometre-sized polystyrene and polymethylmethacrylate particles penetrate both species’ steles through the crack-entry mode at lateral root emergence sites. The efficient absorption of submicrometre plastic is facilitated by this crack-entry pathway and the characteristics of the polymeric particles. Following that, the plastic particles were transferred from the roots to the shoots. This raises obvious concerns, as this process might introduce MPs into the food chain in general, and nectar and pollen specifically. Therefore, additional research is needed to confirm this hypothesis and investigate the potential negative effects of bees being exposed to MPs potentially found in nectar and pollen.

**Figure 1:**
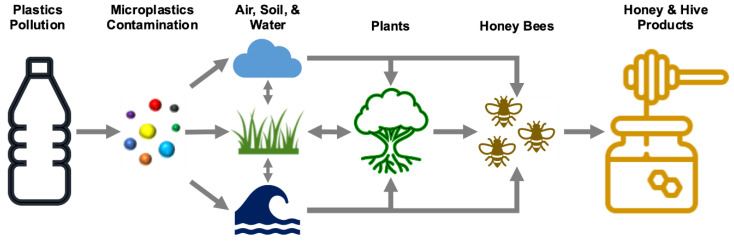
Microplastic particle mass flow in the environment and potential translation into honey bees and other hive products.

Honey bees interact actively with plants, air, soil, and water in the vicinity of the hive, and as a consequence, pollutants from these sources are transferred into honey bees and hive products [45]. Due to their sensitivity and large foraging area, honeybees are, therefore considered to be potential sentinel insect models for monitoring environmental quality [46,47,48]. When honeybees collect nectar, honeydew, pollen, water, and other plant exudates like propolis, they come into contact with almost every environmental compartment. If the nearby compartments are polluted by MPs, they will ultimately be introduced into the honey bee colony and hive products (Figure 1).

In 2013, MPs were observed in honey, which has resulted in considerable attention from both the scientific community as well as the public and news media [49,50]. However, there has been an active debate among researchers concerning these results. Independent researchers were unable to confirm or reproduce these previous results [51]. However, MPs have recently been found in 12% of the honey, beer, milk, and refreshment samples collected in Ecuador, consisting primarily of polyethylene, polypropylene, and polyacrylamide polymers [52]. In addition, MPs have recently been detected in honey bees collected from 19 different apiaries in Copenhagen, as well as suburban and rural areas, primarily as fragments (52%) and fibers (38%) [53]. Expectedly, the highest load was found in urban apiaries; however, more surprisingly, comparable numbers of MPs were also found in suburban and rural hives, which can be explained by the presence of urban settlements within worker bee foraging ranges and the ease with which small MPs can be dispersed by wind [53]. It is also still not clear whether the source of the MPs was due to beekeeping practice or contamination of the beekeeping material introduced into the hive. Since the majority of MPs contained in honey collected from a research apiary in Frankfort, Germany, were fibers, it was thought that they were from beekeepers’ clothes (unpublished data). As a result, detecting MPs directly in nectar samples can aid in determining the sources of MPs found in honey. These findings also suggest that MPs are widely distributed, but the potential induced effects on individual honeybees or other bee species have yet to be investigated. To date, there has been only one study that demonstrated adverse effects of polystyrene (PS)-MPs, under laboratory conditions, on European honey bees (*Apis mellifera* L.). PS-MPs exposure caused almost no survival stress in bees in a 14-day exposure study. Despite this, PS-MP administration reduced diversity of bacteria in the gut and caused major changes in gut microbiomes of bees, as well as changes in gene expression related to oxidative damage, detoxification, and immunity [54]. These sublethal effects of PS-MPs in bees were possibly caused by their aggregation and deterioration in the gut, as well as their subsequent interaction with the microbial population. Alternatively, given that the majority of MPs found in honey and honey bees were irregular fragments and fibers, it is unclear how environmentally representative concentrations, sizes, and shapes of MPs used in the study were [49,50,52,53]. These irregularly shaped materials are widespread in the environment, but they are less commonly considered [55] and they might be more toxic to bees than more spherical equivalents, as shown in previous studies with other invertebrates, such as daphnids [56,57] and aquatic insects [58]. MPs have been shown to cause harm to marine animals, as well as turtles and birds, by blocking digestive tracts, decreasing the desire to eat, and altering feeding behaviour, all of which reduce growth and reproductive performance [59,60]. As a result, future research must consider realistic field exposure scenarios for honey bees to MPs through contaminated honey or pollen, especially fibers and fragments, as well as whether the sizes of plastics found in honey such as fibers: 67.18–3302.68 µm and fragments: 5.63–182.96 µm [52], are small enough to be ingested/internalized by the bees. A study in which bees were fed MPs of different sizes and shapes could provide insight into the response to that question.

MPs may not be the most harmful contaminant, but their toxicity might be greater in the presence of other chemicals. Lethality caused by MPs was significantly increased when the gut microbiota were depleted using the antibiotic tetracycline [54]. Given the reported adverse effects of this antibiotic on fitness of bees [61,62], the dosage used, which was equal to the median lethal dose to bees (LD_50_) and the gut microbial communities of bees that had not been established prior to PS-MPs or/and tetracycline exposure, it is still unclear whether toxicity was caused by the elimination of the gut microbiota or by a synergistic/additive effect of each agent’s toxicity (PS-MPs and tetracycline) [54]. A study in which bees are recolonized shortly after application could provide insight into the response to that question. These findings also call for more research into the combined effects of MPs and other environmental pollutants like heavy metals, pesticides, and nanomaterials, as well as parasites and pathogens, on honey bee health. For example, exposure to MPs led to extensive particle size-dependent gut damage early in life and an enhancement of Cd-induced inhibition of locomotor-behavioral function in adult *Drosophila* flies [63].

Although the above-mentioned study found that exposure to PS-MPs did not result in reduced survival rates in bees [54], lethality is not the only indicator of honey bee colony health [64]. Future research is needed to investigate the potential negative effects on brood pattern, queen egg laying, drone vitality, and colony vigor. On the other hand, it is still also unclear whether MPs accumulate over time and whether this is affected by the type, size, and shape of MPs. Since the life span of worker bees is longer during the winter, accumulation of MPs from contaminated honey may be more important to their survivability and the winter mortality issue.

While methods for assessing ecological risks posed by MPs to terrestrial organisms are still being established, the hazard characterization scheme proposed by the US-EPA’s Office of Chemical Safety and Pollution Prevention for assessing pesticide risks to pollinators could be used [65]. As a consequence, identifying honey bee exposure routes to microplastics and determining concentrations remain crucial. Honey bees can be exposed to MPs when foraging in contaminated fields, as shown in Figure 1. Since, pollen and honey/nectar may represent primary sources of exposure for both larval and adult stages of bees to MPs, both should be included in the investigation. As a result, determining concentrations of MPs in nectar/honey and pollen is essential for better calculating the total daily intake (TDI) based on the total food consumption rate (TFR) of nectar and pollen for adult worker bees. To measure risk estimates, the lowest tier (Tier I) (screening tool) will be evaluated, which uses conservative exposure assumptions (i.e., assumptions that are likely to overestimate exposure) and the most sensitive toxicity estimates from laboratory studies of individual bees. To do that, hazard quotients (HQs) for individual MPs will be calculated based on the total daily intake (TDI) of MPs in honey and pollen divided by the LD50 for each MP. If the sum of HQs of individual MPs exceeded the level of concern (LOC) (i.e., a pre-set number (0~1) that the US EPA uses to equate to the measured HQ in ecological risk assessments to decide the degree of potential risk to non-target species and the need for further regulatory action) [65], then higher-tier assessments (Tier II (semi-field) and Tier III (field studies)) that rely on characterization of risk based on measured exposure values and colony-level effects studies would be evaluated to obtain a more realistic measure of the risk of MPs to honey bees. On the other hand, MPs are a heterogeneous class of materials that vary not only in particle properties such as size and shape, but also in chemical composition, which includes polymers, additives, and side products [66]. Thus far, it is unclear whether the plastic additive or the particle itself is the driving factor for microplastic toxicity.

Another concerning issue is that MPs can adsorb pollutants and thus become both a source and a sink for them due to their lipophilicity [67]. Since more of the surface area of MPs is exposed, their chemical reactivity increases, and degradation of MPs into smaller plastic particles can enhance adsorption of contaminants on MPs. Weathering, sunlight, pH, long exposure times, and the hydrophobicity of POPs are all factors that can affect the kinetics of contaminant adsorption to MPs [68]. Polyethylene (PE) MPs, for example, have been reported to be strong carriers of pesticides in agricultural fields [69], potentially exacerbating pesticide exposure effects on bees. Additionally, the role of MPs in pathogen transmission is still a hot topic. As the amount of plastic debris in the environment grows, the possibility of microplastic acting as pathogen vectors is becoming a growing concern [70]. As a result, further research is required to see whether MPs can spread pathogens among honey bees in general and viruses in particular.

To summarize, honey bees are essential pollinators for crops and the preservation of most ecosystems. On the other hand, MPs now have emerged as an alarming environmental pollutant and their prevalence is now widely observed in various ecosystems. However, only one study has shown adverse effects on European honey bees (*Apis mellifera* L.) exposed to polystyrene (PS)-MPs under laboratory conditions. As a result, several research questions still need to be addressed to assess the potential risks posed by MPs to bees as follows:

- The potential negative effects of MPs on brood pattern, queen laying, drone vitality, and colony vigor.
- Uptake and accumulation of MPs in honey bee tissues and whether this is affected by the type, size, and shape of MPs or not?
- The combined effects of MPs and other environmental pollutants like heavy metals, pesticides, and nanomaterials, as well as parasites and pathogens, on honey bee health.
- The potential role of MPs as vector of honey bee pathogens.

## Data Availability

No new data were created or analyzed in this study. Data sharing is not applicable to this article.
